# Transcriptomic, Biochemical, and Morphological Study Reveals the Mechanism of Inhibition of *Pseudopestalotiopsis camelliae-sinensis* by Phenazine-1-Carboxylic Acid

**DOI:** 10.3389/fmicb.2021.618476

**Published:** 2021-03-30

**Authors:** Qiaoxiu Yin, Rui Yang, Yafeng Ren, Zhiying Yang, Tao Li, Honglin Huang, Qin Tang, Dongxue Li, Shilong Jiang, Xian Wu, Delu Wang, Zhuo Chen

**Affiliations:** ^1^Key Laboratory of Green Pesticide and Agricultural Bioengineering, Ministry of Education, Guizhou University, Guiyang, China; ^2^College of Agricultural, Guizhou University, Guiyang, China; ^3^College of Forestry, Guizhou University, Guiyang, China

**Keywords:** *Pseudopestalotiopsis camelliae-sinensis*, phenazine-1-carboxylic acid, antifungal activity, transcriptome, ultrastructure, exopolysaccharides, action mechanism

## Abstract

Gray blight disease is one of the most destructive diseases of tea plants and occurs widely in the tea-growing areas of the world. It is caused by several fungal phytopathogens, of which *Pseudopestalotiopsis camelliae-sinensis* is the main pathogen in China. The environmentally friendly antimicrobial, phenazine-1-carboxylic acid (PCA), a metabolite of the natural soil-borne bacteria *Pseudomonas* spp., can inhibit a range of fungal crop diseases. In this study, we determined that PCA was active against *Ps. camelliae-sinensis in vitro.* We studied the mode of action of PCA on hyphae using a microscopic investigation, transcriptomics, biochemical methods, and molecular docking. The results of scanning and transmission electron microscopy indicated that PCA caused developmental deformity of mycelia and organelle damage, and it significantly decreased the accumulation of exopolysaccharides on the hyphal surface. The transcriptome revealed that 1705 and 1683 differentially expressed genes of *Ps. camelliae-sinensis* treated with PCA were up-regulated or down-regulated, respectively, with genes associated with ribosome biogenesis, oxidative phosphorylation, and encoding various proteins of *N*-glycan biosynthesis being significantly up-regulated. Up-regulation of nine genes related to *N*-glycan biosynthesis of *Ps. camelliae-sinensis* in response to PCA treatment was confirmed by reverse transcription qPCR. The enzymatic activity of catalase and superoxide dismutase of hyphae was significantly decreased by PCA treatment. Our results indicated that exposure to PCA resulted in expression changes in oxidoreductase genes, accumulation of reactive oxygen species, and decreased activity of catalase, with concomitant damage to the fungal cell membrane and cell wall.

## Introduction

Tea [*Camellia sinensis* (L.) Kuntz] is an economically important crop, the leaves of which are processed into beverages. A perennial evergreen small tree or shrub, tea grows in plantations in tropical and subtropical regions (Li et al., 2019). There are many diseases of tea foliage, which are divided into leaf blight, leaf spot, leaf rot, and leaf rust (Shigeo, 1978; Sanjay et al., 2008; Bao et al., 2019). Tea foliage diseases often damage both the productivity and the quality of the tea leaf (Mukhopadhyay et al., 2016). Gray blight disease is one of the most destructive diseases on tea plants and occurs widely in the tea-growing regions of the world (Horikawa, 1986; Joshi et al., 2009). The closely related fungal phytopathogens *Pestalotiopsis camelliae*, *Pestalotiopsis lushanensis*, *Neopestalotiopsis clavispora*, *Neopestalotiopsis ellipsospora*, *Pseudopestalotiopsis camelliae-sinensis*, and *Pseudopestalotiopsis chinensis* can all cause tea gray blight disease (TGB), and *Ps. camelliae-sinensis* is known to be the dominant pathogen for this disease in China (Maharachchikumbura et al., 2014; Chen et al., 2017, 2018a,b; Liu et al., 2017). Currently, chemical fungicides are the main strategy to prevent and control the disease, but the problem of increased risks of pesticide residues in tea leaves has attracted negative attention (Horikawa, 1986; Oniki et al., 1986; Shin et al., 2000; Tomihama et al., 2009; Yamada et al., 2016). The biocontrol agents, *Trichoderma harzianum*, *Gliocladium virens*, and *Pseudomonas fluorescens*, have been used to prevent and effectively manage TGB (Sanjay et al., 2008). These results suggested that applying the biocontrol agents or the respective active antimicrobial factors could be a promising strategy for control of TGB (Sanjay et al., 2008; Premkumar et al., 2012; Maharachchikumbura et al., 2014).

Phenazines are heterocyclic nitrogen-containing secondary metabolites produced by *Pseudomonas* spp., which have great potential as antimicrobials against various plant fungal pathogens, such as *Gaeumannomyces graminis* var. *tritici*, *Fusarium oxysporum*, *Pythium* spp., *Rhizoctonia solani*, *Gibberella avenacea*, *Alternaria* spp., and *Drechslera graminea* (Mavrodi et al., 2006; Price-Whelan et al., 2006). Phenazine-1-carboxylic acid (PCA) was isolated and identified as a metabolite of the bacterium *Pseudomonas chlororaphis* subsp. *aureofaciens* strain M71, which had been shown to inhibit the growth of the fungal phytopathogens *Alternaria alternata*, *Botrytis cinerea*, *F. oxysporum* f. sp. *radicis-lycopersici*, *G. graminis* var. *tritici*, *Mycosphaeralla melonis*, and *Phellinus noxius* (Brisbane et al., 1989; Puopolo et al., 2013; Huang et al., 2016) *in vitro* and *in vivo*. Treatment with PCA also inhibited growth of the bacterial phytopathogen *Xanthomonas oryzae* pv. *oryzae* (Xoo), causing decreased activity of the antioxidant enzymes catalase (CAT) and superoxide dismutase (SOD), and resulting in the accumulation of reactive oxygen species (ROS; Xu et al., 2015). PCA has also been reported to decrease SOD activity, generate higher intracellular ROS concentrations, inhibit the formation of exopolysaccharides (EPS), and affect the cellular redox homeostasis of *B. cinerea*, *P. noxius*, and *Phoma segeticola* var. *camelliae* (Huang et al., 2016; Simionato et al., 2017; Zhao et al., 2018). In the current study, the mode of action of PCA against *Ps. camelliae-sinensis* was explored through morphology, biochemical studies, RNA-Seq, and reverse transcription qPCR (RT-qPCR) studies.

## Materials and Methods

### Sensitivity of Mycelial Growth of *Ps. camelliae-sinensis* Strain GZHS-2017-010 to PCA

*Pseudopestalotiopsis camelliae-sinensis* strain GZHS-2017-010 was isolated and identified from tea leaves in Huishui County, Guizhou Province, China, and deposited in China General Microbiological Culture Collection Center, with the preservation number of fungus as CGMCC3.20151. PCA (purity of 95%) was purchased from Ark Pharm Inc., IL, United States. Mycelial plugs (6-mm diameter) from the leading edge of a 5-day-old colony of the strain GZHS-2017-010 on potato dextrose agar (PDA) was placed on a series of PDA plates containing 0.0 (control), 2.5, 5.0, 7.5, 10.0, 12.5, or 15.0 μg/ml of PCA, and the PCA was dissolved in dimethyl sulfoxide (DMSO) to a final working concentration of 0.5% (v/v). For each concentration, five replicates were conducted. The diameter (minus the diameter of the inoculation plug) of each colony was measured after incubation for 5 days at 25°C in darkness. The growth inhibition rate expressed as a percentage of the control colony diameter was calculated by the equation:

I(%)=[(C-T)/(C-0.6)]×100

Where *I* is the inhibition rate, *C* is the diameter of the colony in the control group, and *T* is the colony diameter of the PCA-treatment group.

The effective concentration giving 10% inhibition (EC_10_), 30% inhibition (EC_30_), 50% inhibition (EC_50_), 70% inhibition (EC_70_), and 90% inhibition (EC_90_) were calculated based on linear regression of colony diameter vs. log-transformed fungicide concentration. The experiment was independently conducted three times.

### Effect of PCA on Hyphal Morphology of *Ps. camelliae-sinensis*

Mycelial plugs (6-mm diameter) from the leading edge of a 5-day-old colony of the strain GZHS-2017-010 on PDA were transferred to 250-ml flasks of potato dextrose broth (PDB) and cultured on a shaking incubator at 28°C, 180 rpm for 2 days, at which PCA was added to a final concentration of 11.23 μg/ml (EC_50_) and a DMSO working concentration of 0.5% (v/v). The control treatment (CK) contained 0.5% DMSO without PCA. 12 h later, hyphal morphology was observed under optical microscopy (BX43F; Olympus, Tokyo, Japan).

For scanning electron microscopy (SEM), mycelial plugs (6-mm diameter) from the leading edge of 5-day-old colonies of the strain GZHS-2017-010 were transferred to PDA plates containing PCA at 0.00 (control), 6.38 (EC_10_), or 11.23 μg/ml, each with a working concentration of 0.5% DMSO. After 5-day culture at 25°C in darkness, 6-mm mycelial plugs were fixed with 2% glutaraldehyde (0.1 mol/L PBS, pH 7.3–7.4) at 4°C overnight and washed with 0.1 M phosphate buffer saline (PBS; pH 7.4) three times, each for 5 min. Subsequently, the samples were dehydrated in an ethanol series (50, 70, 80, 90, 95, 100, and 100%) for 15 min, each three times. The dehydrated samples were transferred to ethanol:isoamyl acetate (v/v: 50%), then 100% isoamyl acetate for 15 min, and then critical-point dried (K850 Critical-Point Dryer; Quorum Technologies, Lewes, United Kingdom). The morphology of the hyphae was observed by SEM (U8010; Hitachi, Tokyo, Japan).

For transmission electron microscopy (TEM), the fixed mycelial plug samples were washed with 0.1 M PBS three times for 15 min each. Subsequently, the samples were dehydrated in an ethanol series (50, 70, 80, 90, 95, 100, and 100%) three times for 15 min each. The plugs were transferred to a 1:1 mixture of acetone:812 embedding resin [Epon 812 (9.5 ml), MNA (5.6 ml), DDSA (4.9 ml), and DMP-30 (0.3 ml)] and allowed to penetrate overnight, before transfer to 100% 812 embedding agent, and allowing to penetrate overnight. After polymerization at 60°C for 48 h, a microtome was used to produce 60- to 80-nm ultra-thin slices with a diamond knife (UC7; Leica, Wetzlar, Germany). After using uranyl acetate and lead citrate to increase the contrast, samples were observed under TEM (H-7650; Hitachi, Tokyo, Japan).

### Transcriptome Analysis

#### RNA Extraction and Transcriptome Sequencing

Mycelium treated with PCA (final concentration: 11.23 μg/ml) and 0.5% DMSO for 12 h was ground into a fine powder in the presence of liquid nitrogen. About 100 mg of the powder was used for RNA extraction. Total RNAs were extracted using the TRIzol reagent (Invitrogen, Carlsbad, CA, United States). RNA purity was checked using a NanoPhotometer^®^ spectrophotometer (IMPLEN, CA, United States) and RNA concentration was measured using Qubit^®^ RNA Assay Kit and a Qubit^®^ 2.0 Fluorometer (Life Technologies, CA, United States). RNA integrity was assessed using the RNA Nano 6000 Assay Kit of the Bioanalyzer 2100 system (Agilent Technologies, Inc., Santa Clara, CA, United States). RNA degradation and contamination were monitored by electrophoresis on 1% agarose gels. A total amount of 3 μg RNA per sample was used for the RNA sample preparations. Sequencing libraries were generated using the NEBNext^®^ UltraTM RNA Library Prep Kit for Illumina^®^ (New England BioLabs, NEB, Ipswich, MA, United States) following the manufacturer’s recommendations, and index codes were added to attribute sequences to each sample. Briefly, mRNA was purified from the total RNA using poly-T oligo-attached magnetic beads. Fragmentation was carried out using divalent cations in 5× Fragmentation Buffer under elevated temperature in NEB Next First-Strand Synthesis Reaction Buffer (5×). First-strand cDNA was synthesized using random hexamer primers and M-MuLV Reverse Transcriptase (RNase H^–^). Second-strand cDNA synthesis was subsequently performed using DNA polymerase I and RNase H. Remaining overhangs were converted into blunt ends via exonuclease/polymerase activities. After adenylation of the 3’ ends of the DNA fragments, NEBNext adaptors with hairpin loop structures were ligated to prepare for hybridization. In order to select cDNA fragments within the range 250–300 bp in length, the library fragments were purified with the AMPure XP system (Beckman Coulter, Beverly, MA, United States). An aliquot (3 μl) of USER Enzyme (NEB, Ipswich, MA, United States) was used with size-selected, adaptor-ligated cDNA at 37°C for 15 min followed by 5 min at 95°C before PCR was carried out. PCR was performed with Phusion High-Fidelity DNA polymerase, Universal PCR primers and Index (X) Primer. Finally, PCR products were purified (AMPure XP system) and library quality was assessed on the Bioanalyzer 2100 system (Agilent Technologies, CA, United States).

The clustering of the index-coded samples was performed on a cBot Cluster Generation System, using the TruSeq PE Cluster Kit v3-cBot-HS (Illumina, San Diego, CA, United States), according to the manufacturer’s instructions. After cluster generation, the library preparations were sequenced on an Illumina HiSeq platform 4000 (Illumina, San Diego, CA, United States) and 125 to 150-bp paired-end reads were generated.

#### Sequence Assembly and Annotation

Raw data (raw reads) were initially processed using Cutadapt^1^. In this step, clean data (clean reads) were obtained by removing reads containing adaptors, reads containing poly-*N*, and low-quality reads (reads with *Q*_*phred*_ ≤ 20 base number accounting for more than 50% of the whole read length) from the raw data. At the same time, Q20, Q30, and GC content of the clean data were determined. All the downstream analyses were based on the clean high-quality data.

The reference genome (*Pestalotiopsis fici*; Bioproject: PRJNA174299) and gene model annotation files were downloaded from the genome website directly. The index of the reference genome was built using HISAT2 version 2.0.5^2^ and paired-end clean reads were aligned to the reference genome (Kim et al., 2015). We selected HISAT2 as the mapping tool because it can generate a database of splice junctions based on the gene model annotation file and thus achieve a better mapping result than other non-splice mapping tools (Mortazavi et al., 2008).

#### Gene Expression Analysis

The algorithm featureCounts version 1.5.0-p3 was used to count the reads number mapped to each gene, and the number of fragments per kilobase of transcript per million mapped reads (FPKM) of each gene was calculated, based on the length of the gene, and the reads count mapped to this gene (Liao et al., 2014).

Differential expression analysis between two treatment groups (PCA vs. control) was performed using the DESeq2 R package version 1.16.1. DESeq2 provides statistical routines for determining differential expression in digital gene expression data, using a model based on the negative binomial distribution. The resulting *P* values were adjusted using the Benjamini and Hochberg’s procedure for controlling the false discovery rate (FDR). Genes with log2(Fold Change) > 0 and an adjusted *P* value < 0.05, determined by DESeq2, were assigned as being differentially expressed genes (DEGs; Love et al., 2014).

#### GO and KEGG Annotation of DEGs

Gene Ontology (GO) terms^3^ with corrected *P* values less than 0.05 were considered to be significantly enriched with DEGs by the clusterProfiler R package version 3.4.4. Kyoto Encyclopedia of Genes and Genomes (KEGG)^4^ version 2016.05 was implemented by clusterProfiler R package version 3.4.4.

### Validation of the Transcriptome Sequence

In order to validate the DEGs identified from transcriptome sequencing, qPCR analysis was performed for two treatment groups (PCA vs. control). Total RNAs were extracted as described before to obtain the samples used in transcriptome sequencing. Using the PrimeScript RT reagent qPCR Kit with gDNA Eraser (Takara, Dalian, China), genomic DNA was removed from total RNA (300 ng RNA of each sample), and cDNA was synthesized. The PCR mixture contained 10 μl of EvaGreen 2 qPCR MasterMix-No dye [Applied Biological Materials (ABM) Inc. Vancouver, Canada], 7.4 μl of ddH_2_O, 0.8 μl of each gene-specific primer (10 μM), and 1 μl of cDNA template. The qPCR assays were performed in an Applied Biosystems 7500 Real-Time PCR System (ABI, Waltham, MA, United States), with the following program: 95°C for 10 min, 40 cycles of 94°C for 15 s, and 60°C for 30 s. The actin gene was used to normalize the expression levels of the target genes. The relative expression levels of the target genes were calculated by the 2^–ΔΔCT^ method (Livak and Schmittgen, 2001). The primers were designed using Primer 3 software version 4.1.0^5^ and were synthesized by Sangon Biotech (Shanghai) Co., Ltd., Shanghai, China (Supplementary Table 1).

### Gene Expression of the Genes Related to *N*-glycan Biosynthesis

In order to verify the expression trends of the genes related to *N*-glycan biosynthesis, which had been identified by transcriptome sequencing, nine genes were selected from the pathway of *N*-glycan biosynthesis, and their expression status in the hyphae of *Ps. camelliae-sinensis* strain GZHS-2017-010 treated with different concentrations of PCA during different treatment periods (Supplementary Table 2) was determined, using the qPCR methodology described above.

### Effect of PCA on ROS Accumulation in *Ps. camelliae-sinensis* Hyphae

Intracellular ROS accumulation was measured with 2’,7’-dichlorodihydrofluorescein diacetate (H_2_DCFDA; purity of 99.82%; MedChemExpress, Monmouth Junction, United States). The mycelial plugs (6-mm diameter) from the leading edge of a 5-day-old colony of *Ps. camelliae-sinensis* strain GZHS-2017-010 on PDA was transferred to PDB medium and then cultured for 2 days on a shaking incubator at 28°C at 180 rpm. PCA was dissolved in DMSO and then added into the PDB at dosages of 6.38, 11.23, and 16.08 μg/ml. After 12 h culture, the mycelium was collected and washed three times with PBS buffer and then incubated with 5 μg/ml H_2_DCFDA at 30°C for 30 min in darkness. After washing with PBS buffer three times, the hyphae were observed by fluorescence microscopy (FVMPE-RS; Olympus, Tokyo, Japan).

### Effect of PCA on Activity of CAT and SOD of *Ps. camelliae-sinensis*

Catalase and SOD test kits were purchased from Suzhou Keming Biotechnology Co., Ltd. After mycelium was cultured in the presence of different dosages of 6.38, 8.81, 11.23, 13.66, and 16.08 μg/ml of PCA for 12 h and 18 h, the mycelium was collected by filtration. The enzymatic activity was detected with CAT or SOD assay kits, following the manufacturers’ procedures (Suzhou Comin Biotechnology Co., Ltd, Suzhou, China).

### Effect of PCA on EPS Content of *Ps. camelliae-sinensis*

The quantity of EPS produced by the *Ps. camelliae-sinensis* strain GZHS-2017-010 was tested by the previous described method with some modifications (Dubois et al., 1956; Rao and Pattabiraman, 1989; Hou et al., 2019). For preparation of an EPS standard curve, the reaction mixture consisted of 1 ml of a glucose solution (0, 10, 20, 30, 40, 50, and 60 μg/ml), 0.5 ml of a 6% phenol solution, and 2.5 ml of concentrated H_2_SO_4_, vortex 2 or 10 s, incubated for 20 min at 25°C. Absorbance of the solution was measured at 490 nm. A standard curve was generated by plotting absorbance against glucose concentration. Firstly, mycelial plugs (5 mm in diameter) from the margin of 5-day-old colonies on PDA were transferred to LB with 10 ml at 28°C for 2 days for stationary culture. Hyphae were transferred to semisynthetic medium (60 *g* glucose, 12 *g* yeast extract, 4 *g* polypeptone, 1 *g* MgSO_4_⋅7H_2_O, 1 *g* K_2_HPO_4_, and 0.7 *g* MnSO_4_⋅H_2_O) with 80 ml and placed on a rotary shaker (180 rpm, 28°C). After 12 h, partial flasks were amended with PCA at the ultimate concentration of 0.00, 6.38, 8.81, 11.23, 13.66, and 16.08 μg/ml. The flasks were shaken for an additional 12 h. The contents were centrifuged at 10,000 rpm for 20 min, and the supernatants were collected. EPS was precipitated from 50 ml of each supernatant with four volumes of absolute ethanol and then dried. The crude EPS with 40 mg were dissolved in 100 ml of distilled water and quantified with the standard curve. Sterile-distilled water was used as a control. There were three replications for each treatment, and the test was repeated three times.

### Molecular Docking of PCA and Proteins Involved in *N*-glycan Biosynthesis

The DNA sequences identified as DEGs were translated into protein sequences and screened for in the UniProt database, with BLAST. The target sequence was searched with BLAST against the primary amino acid sequence contained in the SWISS-MODEL template library (Camacho et al., 2009; Bienert et al., 2017). Models were built, based on the target-template alignment, using ProMod3^6^ (Waterhouse et al., 2018). The homology models of the proteins were obtained, and the potent binding pockets of these models were predicted using fpocket^7^ (Schmidtke et al., 2011). PCA was docked into these pockets with AutoDock vina^8^ and the binding free energy was calculated using the MM/PBSA method (Trott and Olson, 2010; Gnheden and Ryde, 2015).

### Statistical Analysis

SPSS version 11.5 (SPSS Inc., United States) was used for the statistical analyses of the data on measured the activity of CAT and SOD. ANOVA (least significant difference method) was performed to analyze the differences among the treatment groups (Ma, 2005).

## Results

### Anti-fungal Activity of PCA Toward Mycelial Growth of *Ps. camelliae-sinensis*

**FIGURE 1:**
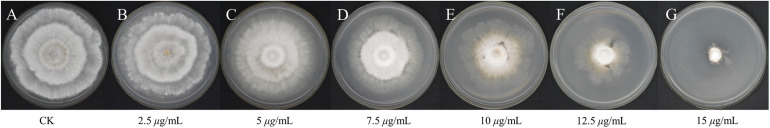
Mycelial growth of *Ps. camelliae-sinensis* strain GZHS-2017-010 treated with different concentrations of phenazine-1-carboxylic acid (PCA). **(A)** CK; **(B)** 2.5 μg/mL; **(C)** 5 μg/mL; **(D)** 7.5 μg/mL; **(E)** 10 μg/mL; **(F)** 12.5 μg/mL; **(G)** 15 μg/mL.

To test the antifungal activity of PCA against the hyphal growth rate *in vitro* of *Ps. camelliae-sinensis* strain GZHS-2017-010, the mycelial growth rate was measured at PCA concentrations of 0.0 (control), 2.5, 5.0, 7.5, 10.0, 12.5, and 15.0 μg/ml, and significant inhibition was apparent (*P* < 0.05). The linear regression equation of mycelial growth rate (*Y*) vs. PCA concentration (*X*) was *Y* = 0.0825*X*-0.4267, *R*^2^ = 0.9853, and EC_10_, EC_30_, EC_50_, and EC_90_ values obtained from the linear regression equation were 6.38, 8.81, 11.23, and 16.08 μg/ml, respectively (Figure 1). This result indicated that PCA could significantly inhibit the growth of hyphae of *Ps. camelliae-sinensis* strain GZHS-2017-010 *in vitro*.

### Effect of PCA on Mycelial Morphology of *Ps. camelliae-sinensis*

**FIGURE 2:**
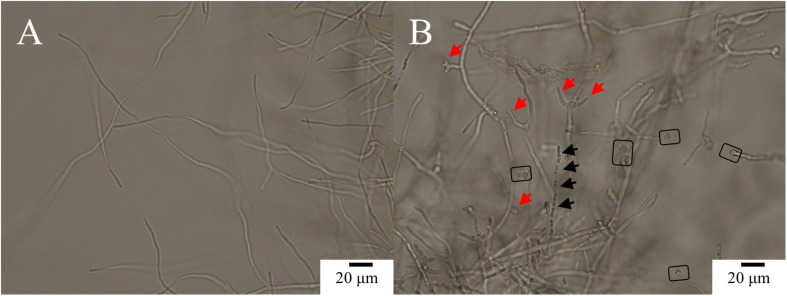
Morphological effects of phenazine-1-carboxylic acid (PCA) on *Ps. camelliae-sinensis* strain GZHS-2017-010 on PDA by optical microscopy. **(A)** Hyphae in the control group; **(B)** Hyphae treated with 11.23 μg/ml PCA for 12 h. Red arrow in **B** indicates hyphal deformity, and a rectangle highlights inflated hyphal tips.

The control (PCA-untreated) hyphae of *Ps. camelliae-sinensis* strain GZHS-2017-010 were smooth, and the development of fresh hyphae, septa, and cell walls was normal (Figure 2A). After the mycelium of *Ps. camelliae-sinensis* strain GZHS-2017-010 was treated with PCA at the EC_50_ concentration of 11.23 μg/ml for 12 h, the hyphae became distorted, and newly formed hyphae developed deformities and grew poorly (Figure 2B, red arrows), with the end of each freshly formed hypha becoming inflated (Figure 2B, rectangles). The cytoplasm condensed to form granulations in the hyphae (Figure 2B, black arrows).

**FIGURE 3:**
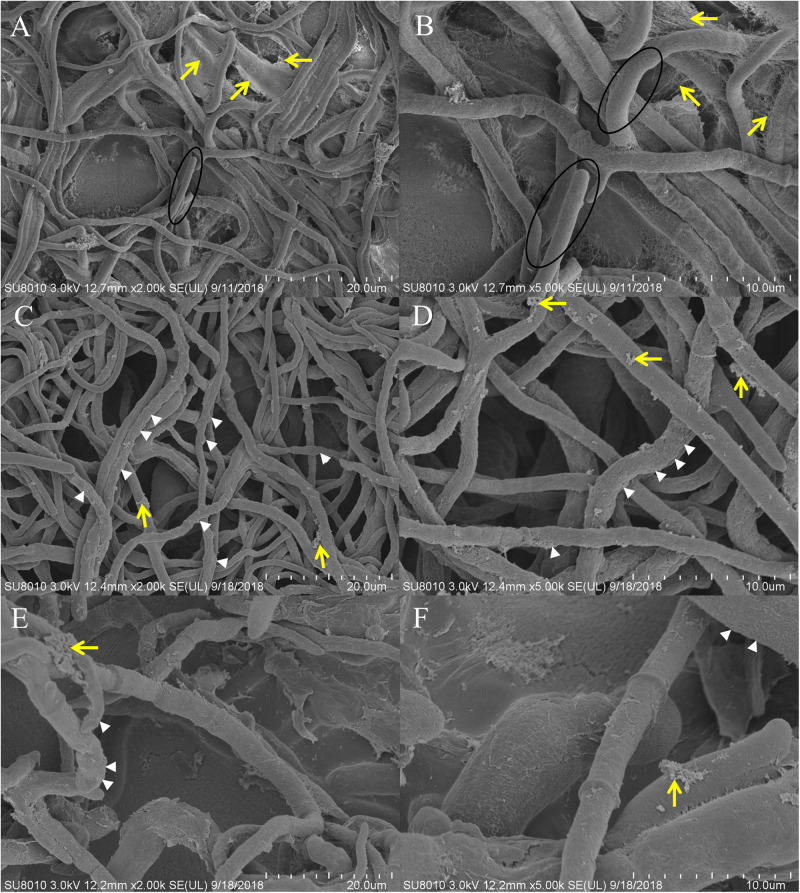
Scanning electron microscopy (SEM) observation of *Ps. camelliae-sinensis* strain GZHS-2017-010 treated with phenazine-1-carboxylic acid (PCA). **(A,B)** Control hyphae; **(C,D)** PCA-treated hyphae at the concentration of 6.38 μg/ml for 120 h; and **(E,F)** PCA-treated hyphae at the concentration of 11.23 μg/ml for 120 h. The magnification levels are shown on each image. The circle in **A,B** indicates normal plump hyphae, of uniform diameter, with the yellow arrows indicating accumulation of exopolysaccharides (EPS). The white arrows in **C–F** indicates the rough hyphal surface and inflated tips of the hyphae, whereas the yellow arrows indicate reduced accumulation of EPS.

Scanning electron microscopy indicated that the control hyphae exhibited morphology characteristic of the species, with a smooth surface, and a uniform and consistent diameter, plump in shape (Figures 3A,B, circled). A large amount of EPS adhered to the surface of the hyphae (Figures 3A,B, yellow arrows). After *Ps. camelliae-sinensis* strain GZHS-2017-010 had been exposed to PCA at 6.38 μg/ml for 120 h, the hyphae exhibited abnormal shapes, with the hyphae being inflated, especially at the ends, with a rough surface (Figures 3C,D, white arrows), and less EPS adhered to the surface of the hyphae (Figures 3C,D, yellow arrows). When the concentration of PCA was increased to 11.23 μg/ml, the hyphae developed serious deformities, with a rough surface (Figures 3E,F, white arrows). The content of EPS was further decreased, compared with the hyphae exposed to the lower PCA concentration (Figures 3E,F, yellow arrows).

**FIGURE 4:**
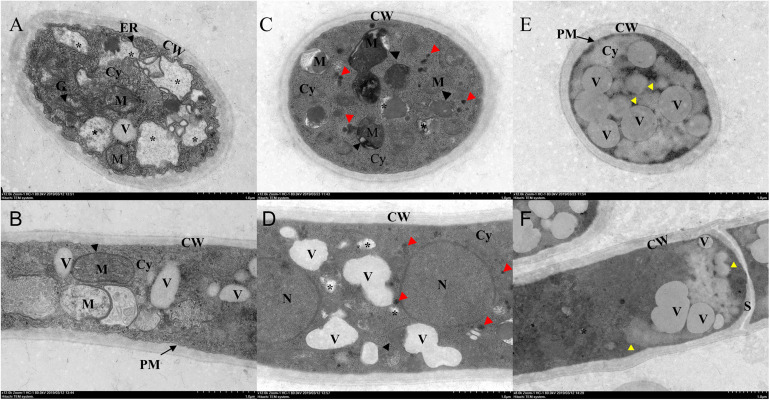
Transmission electron microscopy (TEM) observation of *Ps. camelliae-sinensis* strain GZHS-2017-010 treated with phenazine-1-carboxylic acid (PCA). **(A,B)** Control hyphae, with black arrows indicating endoplasmic reticulum, mitochondria, and Golgi apparatus; **(C,D)** PCA-treated hyphae at the concentration of 6.38 μg/ml for 120 h, with organelles of mitochondria becoming atrophied (black arrows), the cytoplasm condenses into granulation (red arrows) and small cavities (asterisk) in the cytoplasm; and **(E,F)** PCA-treated hyphae at the concentration of 11.23 μg/ml for 120 h, showing serious damage to the organelles (yellow arrows). CW, cell wall; Cy, cytoplasm; ER, endoplasmic reticulum; M, mitochondria; N, nucleus; G, Golgi apparatus; PM, plasma membrane; V, vacuole; and S, septum. Bar = 1 μm.

Transmission electron microscopy showed that the untreated hyphae had normal complete cellular structure and organelles, with clear boundaries to the cell wall, the organellar boundaries of endoplasmic reticulum, mitochondria, and Golgi apparatus (Figures 4A,B). After *Ps. camelliae-sinensis* strain GZHS-2017-010 had been treated with PCA at the concentration of 6.38 μg/ml for 120 h, the boundary structure of the organelles in the mycelium was non-distinct, with organelle of mitochondria having atrophied (Figures 4C,D, black arrows). The cytoplasm had condensed, granulation (Figures 4C,D, red arrows), and small cavities were found in the cytoplasm (Figures 4C,D, asterisk), the volume of the vacuole became large, and the density of the vacuole became lower (Figures 4C,D). When the concentration of PCA was increased to 11.23 μg/ml, organelles in hyphae were seriously damaged and the structure of the organelles was destroyed (Figures 4E,F, yellow arrows). The unclear boundary of the cell wall was obviously changed at the two PCA concentrations (Figures 4C–F), and plasmolysis was represented at the dosage of 11.23 μg/ml of PCA (Figure 4E).

### Effect of PCA on the Transcriptome of *Ps. camelliae-sinensis*

#### Statistics of the Sequences and Their Assembly

In total, 50.99-Gb high-quality sequences were obtained from the transcriptome sequencing of the mycelial samples from the two treatment groups (0.0 and 11.23 μg/ml PCA), ranging from 7.05 to 9.95 Gb per sample (Supplementary Table 3). The average error rates of the sequences were 0.03%. The sequencing data were assembled into 16,699 transcripts with lengths ranging from 150 to 27,639 bases (mean length = 1515 bases and median length = 1272 bases). All RNA-Seq raw datasets were deposited in the NCBI database with a Sequence Read Archive (SRA) accession number of PRJNA579219. For each of the two treatments, the unigenes in the libraries were annotated, using different databases (Supplementary Table 4). Genes were annotated using GO and significantly enriched with respect to the biological, metabolic, and single-organism processes at the level of biological process (BP). At the level of molecular function (MF) aspect, MF, catalytic activity, and binding were significantly enriched. At the level of cellular component (CC) aspect, CC, membrane, and cell were significantly enriched (Supplementary Table 5). The pathways of different metabolic pathways, biosynthesis of antibiotics, ribosomes, biosynthesis of amino acids, and ribosome biogenesis in eukaryotes were significantly enriched using KEGG (Supplementary Table 7).

#### Analysis of DEGs

**FIGURE 5:**
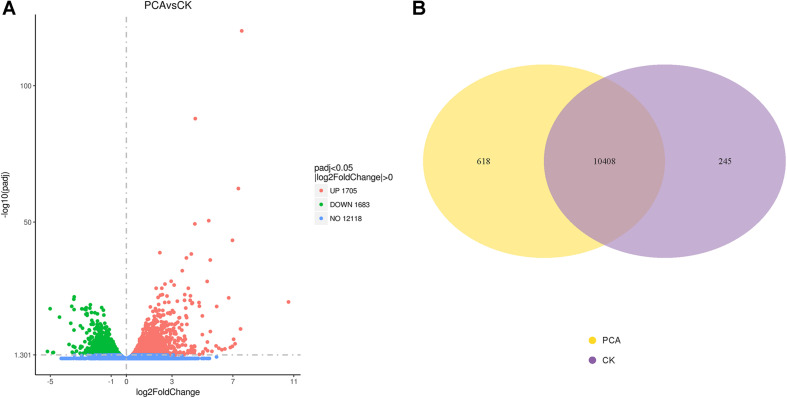
Differentially expressed genes (DEGs) of *Ps. camelliae-sinensis* strain GZHS-2017-010 between PCA-treated hyphae vs. CK-treated hyphae. **(A)** Volcano map of DEGs. In the sub-figure, the *x*-coordinate is log2FoldChange value and the *y*-coordinate is -log10padj; **(B)** Venn diagram of DEGs.

Differential analysis of gene expression was performed on samples from both treatment groups. The RNA-Seq data from PCA-treated vs. CK-treated hyphae revealed that the number of DEGs in *Ps. camelliae-sinensis* strain GZHS-2017-010 was 3388, of which 1705 genes were up-regulated (Figure 5A and Supplementary Table 8) and 1683 were down-regulated in the presence of PCA (Figure 5A and Supplementary Table 9). A total of 323 genes with log2FoldChange values were over 2.0 (Supplementary Table 8), and 100 genes with log2FoldChange values were under -2.0 (Supplementary Table 9). DEGs were regulated by the transcription factors of MFS_1, p450, FAD_binding_8, FAD_binding_3, NAD_binding_1, and NAD_binding_4, etc. using the annotation of tf_family, and the transcription factors represent some difference for DEGs with up-regulation or down-regulation (Supplementary Tables 8, 9). Venn diagrams indicated that 618 and 245 genes were detected for the PCA-treated and control hyphal groups, respectively (Figure 5B and Supplementary Tables 10, 11). A total of 10,408 genes were detected in the two treatment groups (Figure 5B and Supplementary Table 12).

#### GO Enrichment Analysis of DEGs

**FIGURE 6:**
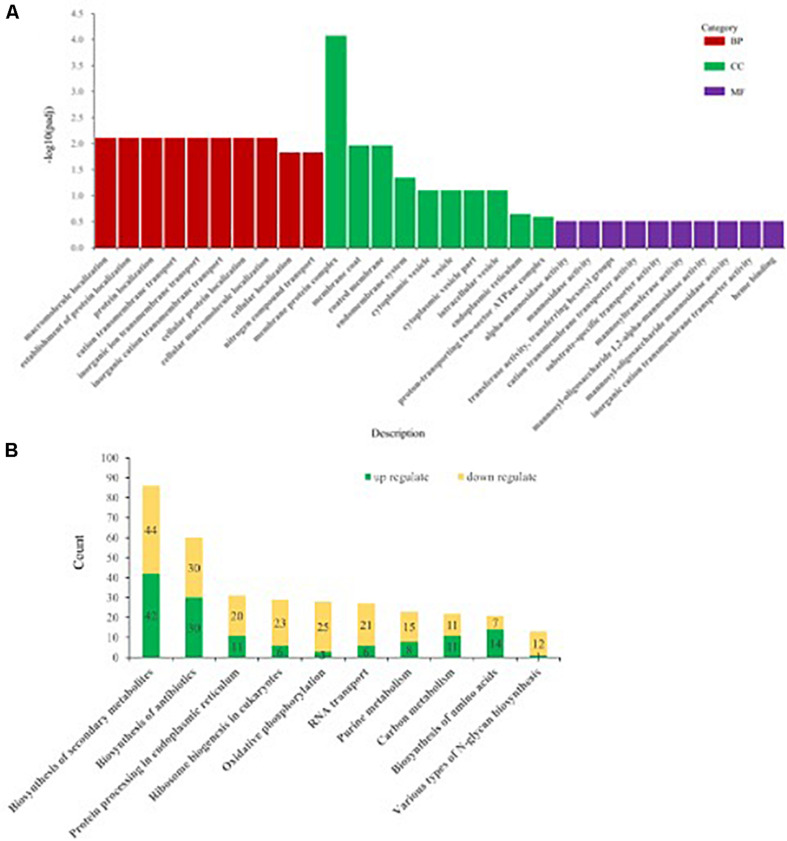
The annotation of differentially expressed genes (DEGs) of *Ps. camelliae-sinensis* strain GZHS-2017-010 between PCA-treated hyphae vs. CK-treated hyphae. **(A)** The annotation of DEGs using Gene Ontology (GO). “BP,” “CC,” and “MF” in the sub-figure represent biological process, cellular component, and molecular function. The *x*-coordinate represent GO term of BP, CC, and MF, and the *y*-coordinate represent -log10padj. **(B)** The annotation of DEGs using Kyoto Encyclopedia of Genes and Genomes (KEGG). The *x*-coordinate represent the description of the pathway, and the *y*-coordinate represent count numbers in the pathway.

Differentially expressed genes of *Ps. camelliae-sinensis* strain GZHS-2017-010 for PCA-treated vs. CK-treated groups were annotated using GO, with count numbers being significantly enriched with respect to the single-organism cellular process, cellular protein metabolic process, and regulation of macromolecule metabolic process at the level of BP being 97, 88, and 72, respectively. At the level of CC aspect, macromolecular complex, cytoplasm, and cytoplasmic part were significantly enriched, with count numbers being 62, 54, and 45, respectively. At the level of MF aspect, transporter activity, oxidoreductase activity (acting on paired donors, with incorporation, or reduction of molecular oxygen), and coenzyme binding were significantly enriched, with count numbers being 96, 87, and 87, respectively (Figure 6A and Supplementary Table 13).

#### KEGG Enrichment Analysis of DEGs

Kyoto Encyclopedia of Genes and Genomes enrichment analysis showed that the DEGs between PCA-treated vs. CK-treated groups were significantly enriched with respect to biosynthesis of secondary metabolites, biosynthesis of antibiotics, protein processing in the endoplasmic reticulum, ribosome biogenesis in eukaryotes, and oxidative phosphorylation, with count numbers being 86, 60, 31, 29, and 28, respectively (Figure 6B and Supplementary Table 14). DEGs with up-regulation or down-regulation in the pathways were annotated, and the ratio of down-regulation was bigger than that of up-regulation (Figure 6B and Supplementary Tables 15, 16). In addition, the pathway of various types of *N*-glycan biosynthesis was significantly enriched, with a count number of 13 (Figure 6B and Supplementary Tables 14–16). Twelve genes were significantly down-regulated in the pathway of various types of *N*-glycan biosynthesis, such as Dolichyl-diphosphooligosaccharide–protein glycosyltransferase subunit stt3, Mannan polymerase II complex ANP1 subunit, and Mannan polymerase complex subunit mnn9 (Supplementary Tables 14–16).

### Validation of DEGs

The expression level of these 11 genes selected from DEGs were verified using qPCR. Gene hypothetical protein (PFICI_14737) was related to *N*-glycan biosynthesis, gene hypothetical protein (PFICI_14203) was related to glycosylphosphatidylinositol (GPI)-anchor biosynthesis, and other genes were not annotated to any particular pathway. qPCR results indicated that 11 genes were regulated in a manner similar to that observed from the transcriptional pattern from the RNA-Seq data (Supplementary Table 15).

### Effect of PCA on ROS Accumulation in *Ps. camelliae-sinensis* Hyphae

**FIGURE 7:**
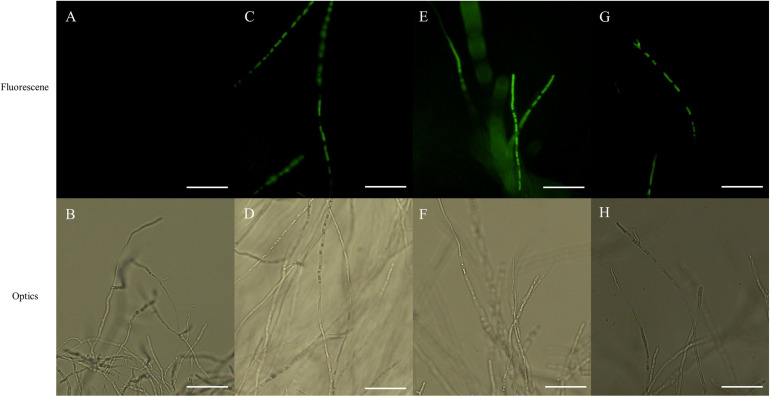
Effects on reactive oxygen species (ROS) accumulation of *Ps. camelliae-sinensis* strain GZHS-2017-010 in response to treatment of hyphae with different concentrations of phenazine-1-carboxylic acid (PCA). **(A,B)** Control hyphae; **(C,D)** PCA-treated hyphae at the concentration of 6.38 μg/ml PCA; **(E,F)** PCA-treated hyphae at the concentration of 11.23 μg/ml PCA; and **(G,H)** PCA-treated hyphae at the concentration of 16.08 μg/ml PCA. Bar = 50 μm. Under fluorescence microscopy, ROS appeared as green fluorescence.

Fluorescence of hyphae, associated with ROS accumulation, was displayed in both PCA-treated and CK-treated hyphae in the presence of H_2_DCFDA, with the intensity of fluorescence being markedly greater in PCA-treated hyphae than in control hyphae, with the intensity of fluorescence increasing with increasing concentration of PCA. The results indicated that ROS accumulated in mitochondria when hyphae were treated with PCA (Figure 7).

### Effect of PCA on Enzymatic Activity in *Ps. camelliae-sinensis* Hyphae

**FIGURE 8:**
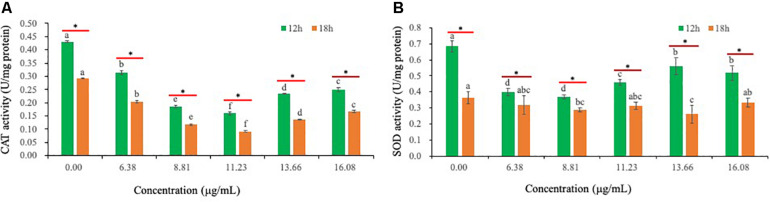
The activities of catalase (CAT) and superoxide dismutase (SOD) of *Ps. camelliae-sinensis* strain GZHS-2017-010 hyphae treated with phenazine-1-carboxylic acid (PCA). **(A)** Effects on CAT activity. Any two samples with shared lowercase letters showed no significant differences, *P* > 0.05; **(B)** Effects of SOD activity. Any two samples with shared lowercase letters showed no significant differences, *P* > 0.05.

Relative to CK-treated hyphae, exposure of *Ps. camelliae-sinensis* strain GZHS-2017-010 hyphae to PCA decreased the activity of CAT in hyphae (Figure 8A). PCA can decrease the activity of SOD, when *Ps. camelliae-sinensis* strain GZHS-2017-010 hyphae was exposed at PCA for 12 h. Nevertheless, the change trend of SOD was not significant when the time of exposure for PCA extended to 18 h (Figure 8B).

### Effect of PCA on *N*-glycan Biosynthesis Gene Expression in *Ps. camelliae-sinensis*

**FIGURE 9:**
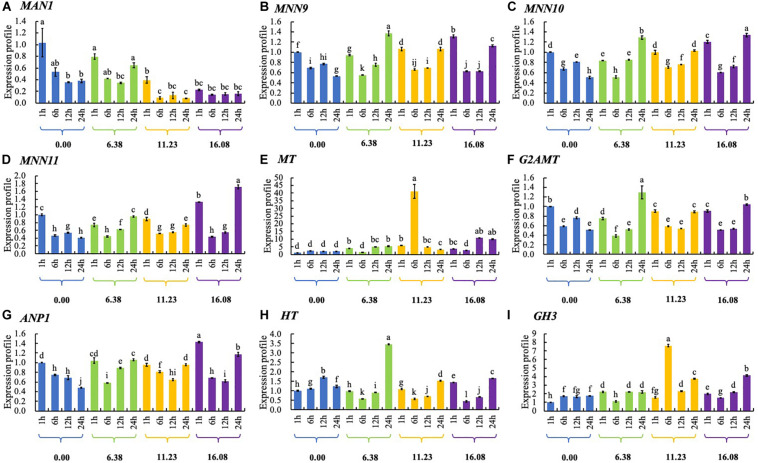
The expression of genes related to *N*-glycan biosynthesis in *Ps. camelliae-sinensis* strain GZHS-2017-010 hyphae treated with phenazine-1-carboxylic acid (PCA). **(A)** *MAN1*: *Mannosyl-oligosaccharide 1,2-alpha-mannosidase*; **(B)** *MNN9*: *Mannan polymerase complexes subunit MNN9*; **(C)** *MNN10*: *Mannan polymerase II complex MNN10 subunit*; **(D)** *MNN11*: *Mannan polymerase II complex MNN11 subunit*; **(E)** *MT*: *Mannosyl transferase*; **(F)** *G2AMT*: *Glycolipid 2-alpha-mannosyltransferase*; **(G)** *ANP1*: *Mannan polymerase II complex ANP1 subunit*; **(H)** *HT*: *Hexosyl transferase*; and **(I)** *GH3*: *Glycoside hydrolase 3*. A shared different lowercase letters showed no significant difference, *P* > 0.05.

In response to increasing PCA concentration, expression of *MAN1* gene was down-regulated in general. With increasing PCA concentration, expression of *mannan polymerase II complex component 9 (MNN9)* gene was up-regulated significantly at the periods of 1-h or 24-h exposure. Expression of *MNN10, MNN11, G2AMT, ANP1, HT, and GH3* genes was up-regulated or down-regulated by PCA at different dosages at different periods. Expression of *MT* gene was significantly up-regulated by PCA at the concentration of EC_50_ during each of the four incubation periods, especially at 6 h (Figure 9).

### Effect of PCA on EPS in *Ps. camelliae-sinensis*

**FIGURE 10:**
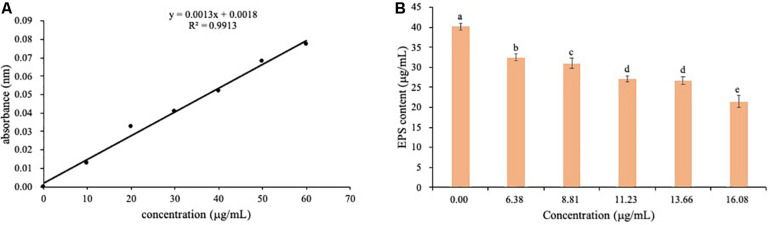
The determination of EPS content of *Ps. camelliae-sinensis* strain GZHS-2017-010 hyphae treated with phenazine-1-carboxylic acid (PCA). **(A)** Standard curve for determination of EPS content. **(B)** Effect of various dosages of PCA on EPS content of *Ps. camelliae-sinensis* strain GZHS-2017-010 hyphae.

The content of EPS was determined by absorbance at 490 nm using the standard curves (Figure 10A). EPS content of *Ps. camelliae-sinensis* strain GZHS-2017-010 treated with 0.00, 6.38, 8.81, 11.23, 13.66, and 16.08 μg/ml PCA showed a downtrend (Figure 10B).

### Molecular Docking of PCA and Proteins Involved in *N*-glycan Biosynthesis

To study the potential targets of PCA, nine proteins were selected for molecular docking studies with PCA. The DNA sequences were translated into protein sequences and run by BLAST in UniProt. Unfortunately, there are no crystal structures available for any of the nine protein sequences. We then used SWISS-MODEL to perform the homology modeling study, and the templates and identities of each protein are shown in Supplementary Table 18.

**FIGURE 11:**
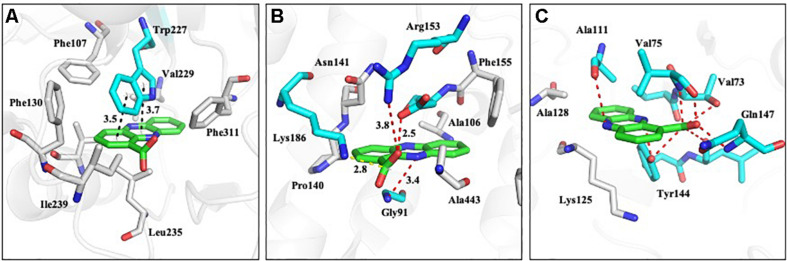
The proposed mode of interaction between PCA and proteins involved in *N*-glycan biosynthesis of *Ps. camelliae-sinensis* strain GZHS-2017-010. The PCA and protein are shown in the purple and green stick model, respectively. The hydrogen bond is shown in black dashed lines. **(A–C)** represent Glycolipid 2-alpha-mannosyltransferase, glycoside hydrolase 3, and Mannan polymerase II complex, respectively.

The homology models of nine proteins were obtained, and the potent binding pockets of these models were predicted using fpocket. The drug was docked into these pockets with a consistent molecular docking by using AutoDock4.2, AutoDock Vina, rDOCK, and LeDOCK. The docking conformation of each software was clustered according to RMDS < 2.0. By voting, the one with the most conformations and the highest comprehensive score is selected as the favorable conformation for the binding energy calculations. The structure of the complex with favorable conformation was used as the starting point for further MD simulation of 3 ns. A total of 100 snapshots of the last 1 ns were selected to calculate the binding free energy using the MM/PBSA method (Table 1). Among these proteins, y3 (Figure 11A), y4 (Figure 11B), and y9 (Figure 11C) were the most potent target with the binding free energy of -37.14, -28.65, and -28.19 kcal/mol, respectively.

**TABLE 1:** The binding free energy (kcal/mol) of drug with nine homology models.

Protein no.	Δ*E*_*vdw*_	Δ*E*_*ele*_	Δ*E*_*PB*_	Δ*E*_*SA*_	Δ*G*
Y1	136.61	–35.66	100.94	–102.84	–1.89
Y2	–27.99	–17.82	–45.82	33.18	–12.63
Y3	–4.84	–39.32	–44.16	7.02	–37.14
Y4	154.54	–35.49	119.05	–147.7	–28.65
Y5	15.64	–14.96	0.69	–12.67	–11.98
Y6	139.13	–22.14	116.98	–125.34	–8.36
Y7	1.55	–27.65	–26.1	10.17	–15.92
Y8	–18.96	–29.13	–48.09	24.7	–23.4
Y9	81.96	–31.74	50.22	–78.41	–28.19

## Discussion

Tea gray blight disease is an important disease in tea plantations and is difficult to prevent or control (Horikawa, 1986; Joshi et al., 2009). It is possible that measures such as microbial pesticides or antimicrobial metabolites of microorganisms could be successful (Sanjay et al., 2008). PCA belongs to the phenazine family of compounds, which have been reported to be effective against many fungi (Brisbane et al., 1989; Puopolo et al., 2013; Huang et al., 2016; Zhao et al., 2018). In a previous study, PCA has been applied in tea plantation to effectively control leaf spot caused by *Didymella segeticola* (Zhao et al., 2018).

In this study, PCA can effectively inhibit the growth of *Ps. camelliae-sinensis*, the main pathogen causing TGB (Figure 1). The DCFH-DA fluorescence assay indicated that ROS was accumulated in *Ps. camelliae-sinensis* treated by PCA (Figure 7), and ROS generation is likely to be an important action mechanism of PCA, which was the validation of PCA treated *X. oryzae* pv. *oryzae*, the phenazines clofazimine treated *Candida albicans* (Morales et al., 2010; Xu et al., 2015). Meanwhile, PCA decreased the activity of SOD and POD in a dose-dependent manner and then reduced ROS scavenging ability (Figure 8). Our results were similar to findings from the PCA treatment of the bacterial phytopathogen *X. oryzae* pv. *oryzae*, which was associated with accumulated ROS and decreased the activity of SOD and CAT (Xu et al., 2015). So, after a large amount of exogenous ROS being accumulated and not cleared by the enzymes, ROS damaged the organellar membranes and the organelles. SEM and TEM also verified that PCA treatment can seriously damage the organellar membranes in hyphae of *Ps. camelliae-sinensis*, changing the structure of the organelles by ROS accumulation (Figures 3, 4). Meanwhile, ROS accumulation induced by PCA can cause the deformity of bacteria (Zhang et al., 2017).

Transcriptome indicated that DEGs were significantly enriched in oxidoreductase activity (acting on paired donors, with incorporation or reduction of molecular oxygen) at the MF level. The transcriptome of *Phytophthora infestans* treated with PCA had also indicated that DEGs related with oxidoreduction activity were significantly enriched (Roquigny et al., 2018). For instance, the gene of eburicol 14 alpha-demethylase was significantly down-regulated by PCA (Supplementary Tables 8, 12). Over-expression of eburicol 14 alpha-demethylase can increase the cytochrome P450 activities in filamentous fungi, and increased resistance of eburicol 14 alpha-demethylase inhibitors in *Aspergillus niger* strains (van den Brink et al., 1996). The result indicated that the decrease of eburicol 14 alpha-demethylase was related with the antifungal activity of PCA.

**FIGURE 12:**
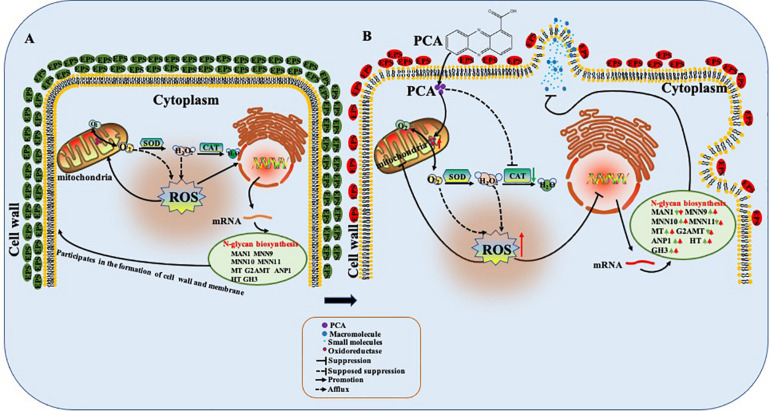
Possible mode of action of phenazine-1-carboxylic acid (PCA) on *Ps. camelliae-sinensis* hyphae. When PCA entered into the hyphal cell, PCA changed oxidoreductase activity, decreased activity of catalase (CAT), and accumulated reactive oxygen species (ROS), so as to induce the deformity of hyphae and inhibit the growth of the hyphae. Meanwhile, PCA influenced the transcription in the pathway of *N*-glycan biosynthesis of hyphae and decreased the content of exopolysaccharides (EPS) on the hyphal surface. **(A)** The untreated hyphae; **(B)** The hyphae treated with PCA.

Phenazine-1-carboxylic acid treatment can decrease the content of EPS of *Ps. camelliae-sinensis* strain GZHS-2017-010, with the inhibition effect being dependent on the dosages (Figures 3, 10). Interestingly, DEGs in transcriptome were also enriched in the pathway of the *N*-glycan biosynthesis, using KEGG annotation (Figure 6). The pathway of the *N*-glycan biosynthesis was involved with the formation of EPS, in which some genes were the key elements of fungus for EPS production (He et al., 2018). GH3 was thought to disrupt the formation of EPS (Wu et al., 2019), and the gene expression of GH3 was up-regulated by PCA (Figure 9). The results were similar to those exhibited by *Pseudomonas solanacearum* and *B. cinerea* treated with PCA (Huang et al., 1995; Simionato et al., 2017). Glycoside hydrolase was involved with the biosynthetic pathway of EPS and possesses the antifungal activity through changing the structure of biofilm (Snarr et al., 2017). The results of molecular docking indicated that PCA possess higher affinity with some proteins in the pathway of *N*-glycan biosynthesis (Table 1 and Figure 11). These results indicated that PCA may bind with the protein in the pathway and then disturb the formation of EPS. We speculate a possible mode of action for PCA as follows (Figure 12). When PCA enters into a hyphal cell, PCA can induce ROS accumulation, result in the deformity of hyphae and thus inhibit the growth of hyphae. Meanwhile, PCA treatment can induce DEGs related with *N*-glycan biosynthesis and decrease the content of EPS on the hyphal surface. Nevertheless, the detailed mechanism of PCA was still unclear. In the future, the action mode between PCA and some proteins from the pathway of *N*-glycan biosynthesis was further studied using multiple methods.

## Conclusion

Phenazine-1-carboxylic acid proved to exhibit antifungal activity *in vitro* toward *Ps. camelliae-sinensis*, the pathogen of gray blight disease. PCA can decrease the activities of CAT and SOD, accumulate ROS, and influence the transcription in the pathway of *N*-glycan biosynthesis, resulting in change of the structure and function of organelles through ROS accumulation, as well as decreasing the content of EPS.

## Data Availability Statement

The datasets generated and/or analyzed in this study are available in the NCBI SRA database with the SRA accession PRJNA579219. SRA records are accessible with the following link: https://www.ncbi.nlm.nih.gov/bioproject/PRJNA579219.

## Author Contributions

ZC conceived and designed the experiments, conducted the whole study, and edited the manuscript. QY and RY performed the experiment of transcriptome and performed the data analysis in this work. YR, ZY, and TL performed the experiment of antifungal bioactivity and the experiment of transcriptome. DL performed the experiment of qPCR. QT, HH, SJ, and XW helped with the data analysis. DW wrote the manuscript. All authors contributed to the article and approved the submitted version.

## Conflict of Interest

The authors declare that the research was conducted in the absence of any commercial or financial relationships that could be construed as a potential conflict of interest.

## Footnotes

1 https://github.com/marcelm/cutadapt/

2 http://www.ccb.jhu.edu/software/hisat/index.shtml

3 http://geneontology.org

4 http://www.kegg.jp/kegg

5 http://bioinfo.ut.ee/primer3-0.4.0/

6 https://openstructure.org/promod3/3.1/

7 https://bio.tools/fpocket

8 https://bio.tools/AutoDock_Vina
